# Effect of Live Poultry Market Closure on Avian Influenza A(H7N9) Virus Activity in Guangzhou, China, 2014

**DOI:** 10.3201/eid2110.150623

**Published:** 2015-10

**Authors:** Jun Yuan, Eric H.Y. Lau, Kuibiao Li, Y.H. Connie Leung, Zhicong Yang, Caojun Xie, Yufei Liu, Yanhui Liu, Xiaowei Ma, Jianping Liu, Xiaoquan Li, Kuncai Chen, Lei Luo, Biao Di, Benjamin J. Cowling, Xiaoping Tang, Gabriel M. Leung, Ming Wang, Malik Peiris

**Affiliations:** Guangzhou Center for Disease Control and Prevention, Guangzhou, China (J. Yuan, K. Li, Z. Yang, C. Xie, Yufei Liu, Yanhui Liu, X. Ma, J. Liu, X. Li, K. Chen, L. Luo, B. Di, M. Wang);; The University of Hong Kong, Hong Kong, China (E.H.Y. Lau, Y.H.C. Leung, B.J. Cowling, G.M. Leung, M. Peiris);; The Eighth People’s Hospital of Guangzhou, Guangzhou, China (X. Tang)

**Keywords:** avian influenza, influenza, respiratory infections, H7N9, live poultry markets, dressed poultry markets, market closure, disinfection, surveillance, retail markets, interventions, control, Guangzhou, Guangdong Province, China, southern China, viruses, zoonoses, human exposure, transmission

## Abstract

We assessed the effect of closing live poultry markets in China on influenza A(H7N9) virus detection and viability. Intensive sampling was carried out before, during, and after a 2-week citywide market closure; the markets were cleaned and disinfected at the beginning of the closure period. Swab samples were collected at different sites within the markets and tested for H7N9 by real-time reverse transcription PCR and culture. During the closure, H7N9 viral RNA detection and isolation rates in retail markets decreased by 79% (95% CI 64%–88%) and 92% (95% CI 58%–98%), respectively. However, viable H7N9 virus could be cultured from wastewater samples collected up to 2 days after the market closure began. Our findings indicates that poultry workers and the general population are constantly exposed to H7N9 virus at these markets and that market closure and disinfection rapidly reduces the amount of viable virus.

## Abstract 2

Temporary closure and disinfection can rapidly reduce levels of infectious virus in these settings.

Influenza A(H7N9) virus emerged in eastern China in March 2013; within 2 years, infections were confirmed in >550 persons and >200 persons had died (*1*). Birds in live poultry markets (LPMs) are considered a major source of H7N9 infection in humans (*2*–*4*). On April 1, 2013, Guangzhou, the capital of Guangdong Province in southern China, implemented surveillance for avian influenza viruses (AIV) in 144 LPMs, in parallel with strengthened surveillance in humans (*5*). Measures included interventions such as daily cleaning, disinfection, and monthly rest days during which poultry were cleared from the markets. Before H7N9 virus infections were identified in humans or poultry in Guangdong Province, the interventions reduced detections of other AIVs by 34% in retail LPMs. (*6*).

When the second epidemic wave of H7N9 virus infection in humans began in October 2013, the virus had spread to China’s southern provinces, and Guangdong Province reported the highest number of infections (*7*). However, in Guangzhou, where the LPM interventions were still in place, no cases of H7N9 virus infections in humans were detected until mid-January 2014 (*8*); by mid-February, the case count reached 10 (*5*). In response, the Guangzhou city administration announced a 2-week citywide market closure starting on February 15, during which trading and storing of live poultry were banned in all locations, including retail and wholesale LPMs (*9*). Only sales of frozen poultry were allowed in supermarkets and malls. The Guangzhou Center for Disease Control and Prevention (GZCDC) established enhanced surveillance in addition to the existing routine LPM surveillance to assess its effect on H7N9 virus isolation and survival.

Previous evidence showed that market closures are highly effective in preventing H7N9 virus infections in humans (*10*,*11*) by substantially reducing human exposure to poultry. However, evidence regarding the effect of such closures on AIV activity within the market environment is limited. Such information may better inform the decision to try alternative interventions, such as market rest days or a ban on keeping unsold poultry in LPMs overnight. We assessed the effect of market closure on virus isolation and survival in a natural LPM setting. The study protocol was reviewed and approved by the Research Ethics Committee of GZCDC.

## Materials and Methods

### Collecting and Testing Environmental Samples

**Figure 1:**
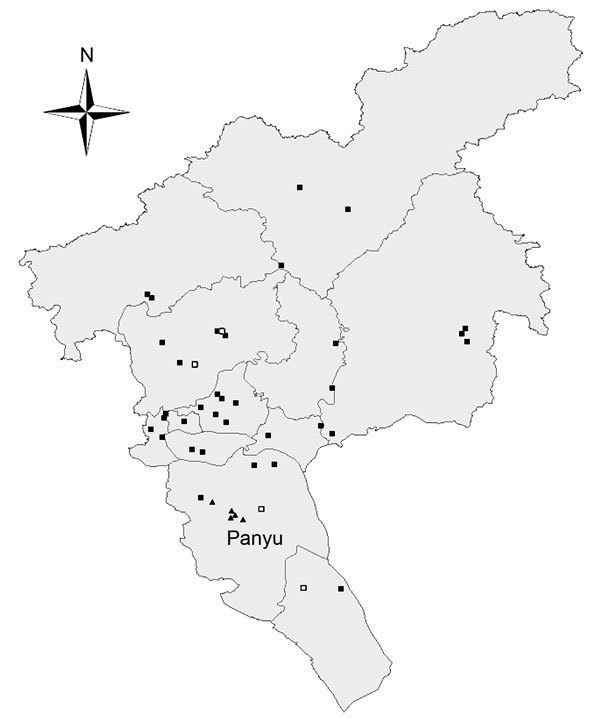
Geographic distribution of the live poultry markets under routine and enhanced surveillance in Guangzhou, China, 2014. Squares indicate routine surveillance sites; solid triangles indicate enhanced surveillance sites (in Panyu district); open squares indicate markets selected for comparison before and after market closure and disinfection.

Routine and enhanced surveillance were established in LPMs in Guangzhou for long-term AIV monitoring and investigation of elevated AIV activity, respectively. Routine surveillance of LPMs was initiated in all 12 districts in Guangzhou on December 26, 2013; a total of 39 randomly selected LPMs were involved. A total of 2–6 retail LPMs from each district and 3 wholesale LPMs from the city were randomly selected; 4–5 environmental swab samples were collected each week from 2–4 randomly selected retail or wholesale stalls. During January 23–30, 2014, additional environmental samples were collected in 4 LPMs immediately before and after poultry were removed and after the markets were disinfected; the samples were tested to assess the effectiveness of the interventions (Figure 1). Swab samples were collected and tested individually.

The citywide LPM closure was implemented during February 15–28, 2014. The LPMs were disinfected once, on February 14, after poultry were removed and the markets thoroughly cleaned. Poultry cages, surfaces of processing tables, and the floor were cleaned with 0.05%–0.1% diluted chlorine solution (expected chlorine concentration 500–1,000 mg/L water). To limit potential market-specific variations in virus activity, enhanced surveillance sites were set up in Panyu district, which comprises one tenth the population of Guangzhou (Figure 1). Samples were collected from retail and wholesale LPMs and from a dressed poultry market (DPM), in which poultry are stocked, processed, and traded differently than they are in LPMs (Table 1). Three retail LPMs, 1 wholesale LPM, and 1 DPM were randomly selected from 77 wet markets in Panyu district. During enhanced surveillance, 12 rounds of intensive sampling were performed before, during, and after the 2-week citywide LPM closure.

**Table 1:** Characteristics of 5 poultry markets under enhanced surveillance, Guangzhou, Guangdong Province, China, 2014*

Market characteristic	Live poultry markets		Dressed poultry market
	Retail	Wholesale	
Source of poultry	Wholesale market	Backyard or large farms	Wholesale market
Volume of poultry stock	Small	Large	Small
Live poultry sold	Yes	Yes	No
Size, m^2^	60/50/50†	3,000	25
No. poultry stalls	6/5/5†	67	5
Approximate no. poultry traded/day	206/285/112†	28,640	190
On-site slaughtering	Yes	Yes	No
Available sampling sites			
Poultry cage	Yes	Yes	No
Defeathering machine	Yes	Yes	No
Chopping board	Yes	No	Yes
Processing table	Yes	No	Yes
Bucket holding poultry meat	No	Yes	No
Wastewater	Yes	Yes	Yes
Poultry drinking water	No	Yes	No
*Three retail LPMs, 1 wholesale LPM, and 1 DPM were randomly selected for study from 77 wet markets in Panyu district, where enhanced surveillance was implemented. †Data are for the 3 retail markets.			

During routine and enhanced surveillance, GZCDC collected environmental samples from poultry cages; the inner surface of defeathering machines; chopping boards; surfaces of processing tables; and barrels holding poultry meat, wastewater, and drinking water for chicken. Because each type of LPM has a different setup for poultry processing and sales, environmental samples were collected only from the relevant sampling sites available within the respective LPMs (Table 1).

### Laboratory Procedures

Universal transport medium (Copan Italia, Brescia, Italy) was used to preserve the environmental samples, which were stored in a box with ice packs at 4°C and transported to the laboratory within 4 hours. A QIAamp Viral RNA Mini Kit (QIAGEN, Hilden, Germany) was used to extract viral RNA. Influenza A virus M gene and H7N9 virus hemagglutinin (HA) RNA were detected as described previously (*12*) by using a real-time reverse transcription PCR (rRT-PCR) (SuperScript III Platinum One-Step qRT-PCR Kit; Invitrogen, Carlsbad, CA, USA) and H7-specific primers and probe provided by the Chinese National Influenza Center. Samples positive for H7N9 virus by rRT-PCR were inoculated into the allantoic sac of 10-day-old specific pathogen free embryonated chicken eggs and incubated for 48–72 h at 35°C for virus isolation (*13*).

### HA Gene Sequencing and Phylogenetic Analysis

The HA gene of isolated strains was amplified by rRT-PCR, and the products were sequenced by Life Technologies Inc. (Carlsbad, CA, USA) as described previously (*14*). The HA sequences were submitted to GenBank (accession nos. KP326319–KP326321). Reference HA sequences were obtained from GenBank from H7N9 virus strains isolated from eastern and southern China. We performed multiple sequence alignments and constructed the phylogenetic tree with MEGA 6.0.6 (http://www.megasoftware.net) by using a neighbor-joining method with 1,000 bootstrap replicates.

### Statistical Analysis

We calculated rRT-PCR detection rates for H7N9 virus only and for all AIVs from the enhanced surveillance before and after disinfection in 4 markets under routine surveillance by dividing the number of rRT-PCR–positive results by the number of samples tested. Samples were screened by rRT-PCR, and those that were H7N9-positive were further tested by culture if sufficient material was available. Therefore, the H7N9-positive isolation rate at each time point was calculated by multiplying the proportion of rRT-PCR–positive samples by the proportion of culture-positive samples. We assumed a binomial distribution and provided exact 2-sided 95% CIs for the detection rates. We obtained the 95% credible intervals for H7N9 virus-positive isolation rates by using a Bayesian method with the Jeffreys noninformative beta distribution priors for the positive proportions by rRT-PCR and by culture.

We tested the effects of market disinfection and closure on detection rates for H7N9 virus and for all AIVs at different times after these interventions were implemented. Logistic regression models were used, accounting for potential confounders such as specific markets and sampling sites. The effect of market disinfection and closure on H7N9 virus isolation rates was similarly tested, after accounting for missing data from H7N9 virus rRT-PCR–positive samples not available for culture, by using multiple imputation methods with 50 imputed datasets. Given the short study period, limited effects of meteorological variables on virus activity were assumed and not adjusted for in the model. We also compared virus detection rates between the different sampling sites. All statistical analyses were conducted by using R version 3.1.1 (https://www.r-project.org/).

## Results

**Figure 2:**
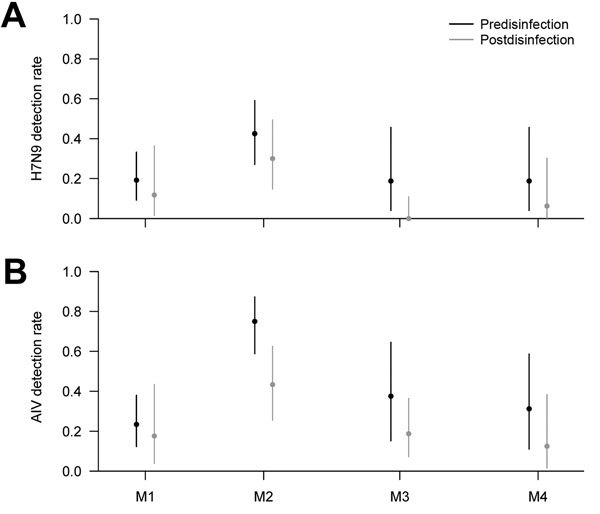
Detection rates for A) avian influenza A(H7N9) virus and B) all avian influenza viruses (AIVs) by using real-time reverse transcription PCR before and after disinfection in 4 retail live poultry markets (M1–M4), Guangzhou, China, 2014.

In the routine surveillance, 214 samples were collected from 4 retail LPMs on the same day immediately before and after the LPMs were disinfected. Testing showed a moderate decrease in the rates of detection of H7N9 virus and other AIVs by rRT-PCR in each LPM after disinfection (Figure 2). The pooled estimated reduction ratios were 58.0% (95% CI 8.9%–80.6%) for H7N9 virus and 64.2% (95% CI 30.6%–81.5%) for all AIVs.

**Figure 3:**
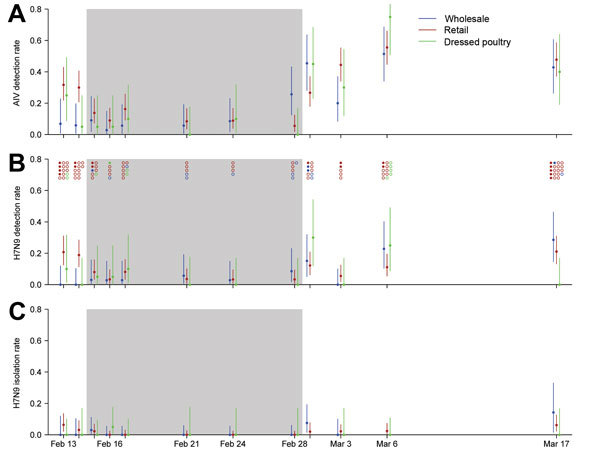
Avian influenza virus (AIV) activity in wholesale, retail, and dressed poultry markets under enhanced surveillance in Guangzhou, China, 2014. A) AIV and B) influenza A(H7N9) virus detection rates as determined by real-time reverse transcription PCR (rRT-PCR). Circles at the top of panel B indicate H7N9 virus–positive (solid) and –negative (open) samples isolated by culture from the different types of poultry markets. Some H7N9 virus samples positive by rRT-PCR did not have sufficient sample remaining for virus culture; the numbers of samples cultured and positive at each time point are shown. C) H7N9 virus isolation rates as determined by culture. Rates for positive cultures were estimated on the basis of the samples available for culture, as described in Materials and Methods. Vertical bars indicate 95% CIs for detection rates and 95% credible intervals for isolation rates. Gray shading indicates the 2-week citywide market closure, which began on February 15, 2014.

A total of 1,705 environmental samples were collected from the targeted enhanced LPM sites. Figure 3, panel A shows the detection rates of AIVs, including H7N9 virus, by rRT-PCR before, during, and after the period of the citywide market closure. Before market closure, site-matched testing (i.e., testing of the same environmental locations between different types of markets) showed the detection rates for H7N9 virus and for all AIVs were significantly higher for retail LPMs than for wholesale LPMs (p = 0.003 and p = 0.032, respectively). Detection rates for AIVs (including H7N9 virus) were higher in retail LPMs than in the DPM (p = 0.043). The samples positive for H7N9 virus by rRT-PCR were cultured for virus isolation (Figure 3, panels B and C).

In the retail LPMs under enhanced surveillance, in the nonintervention period, H7N9 virus isolation rates were <20%, and H7N9 virus and AIV RNA detection rates by rRT-PCR fluctuated at 10%–30% and 20%–60%, respectively. On the first day of market closure, after the markets had been disinfected the preceding night, detection rates for H7N9 virus and all AIVs decreased by ≈50% (Figure 3). During the entire market closure period, RNA detection rates for H7N9 virus and all AIVs decreased by >70% in retail markets, and H7N9 virus isolation rates decreased by >90% (Table 2). After retail LPMs were reopened, H7N9 virus isolation rates increased to a level slightly lower than those before closure.

**Table 2:** Estimated effect of market closure and contaminated environmental sites on AIV and influenza A(H7N9) virus detection in 5 poultry markets under enhanced surveillance, Guangzhou, Guangdong Province, China, 2014*

Variable	Retail LPMs, aOR (95% CI)†				Wholesale LPM, aOR (95% CI)				DPM, aOR (95% CI)	
	rRT-PCR		H7N9 culture		rRT-PCR		H7N9 culture		rRT-PCR	
	AIV	H7N9			AIV	H7N9			AIV	H7N9
Period										
Before market closure	Ref	Ref	Ref		Ref	–‡	–‡		Ref	Ref
During market closure	0.25 (0.16–0.39)	0.21 (0.12–0.36)	0.08 (0.02–0.42)		1.60 (0.52–4.90)	0.22 (0.10–0.50)	0.11 (0.01–0.89)		0.30 (0.09–0.98)	0.68 (0.12–3.89)
After market closure	1.78 (1.20–2.63)	0.58 (0.35–0.95)	0.73 (0.27–1.98)		10.3 (3.52–30.3)	Ref	Ref		5.27 (1.97–14.1)	3.32 (0.68–16.1)
Environmental samples tested										
Poultry cage	Ref	Ref	Ref		Ref	Ref	§		–	–
Defeathering machine	1.15 (0.61–2.14)	1.66 (0.74–3.70)	1.25 (0.20–7.87)		2.49 (1.09–5.68)	1.21 (0.40–3.65)	§		–	–
Chopping board	2.64 (1.60–4.37)	2.12 (1.06–4.26)	3.52 (0.88–14.0)		–	–	–		0.56 (0.22–1.41)	3.18 (0.98–10.3)
Processing table	1.16 (0.73–1.85)	1.15 (0.59–2.25)	1.09 (0.26–4.67)		–	–	–		Ref	Ref
Bucket holding poultry meat	–	–	–		0.97 (0.38–2.44)	0.17 (0.02–1.40)	§		–	–
Wastewater	1.60 (0.95–2.67)	1.23 (0.58–2.62)	1.41 (0.28–7.14)		1.38 (0.70–2.73)	0.91 (0.37–2.22)	§		1.15 (0.44–3.06)	1.16 (0.31–4.36)
Drinking water	–	–	–		2.02 (0.44–9.38)	2.32 (0.40–13.4)	§		–	–

*AIV, avian influenza virus; aOR, adjusted odds ratio; DPM, dressed poultry market; LPM, live poultry market; ref, reference; rRT-PCR, real-time reverse transcription PCR; –, no samples tested. †Also adjusted for potential market differences for the 3 retail markets. ‡No influenza A(H7N9) virus was detected before market closure in wholesale markets, and data from this period were excluded from the regression model. §There were too few H7N9 virus–positive samples by culture in contaminated environmental sites in wholesale markets and DPM overall for us to estimate the effects. A simplified model was used for wholesale markets.

The wholesale market had low H7N9 virus detection and isolation rates before and during market closure, but rates increased markedly once the markets reopened. In the DPM, detection rates for AIVs decreased substantially during the market closure and increased greatly when the market reopened. In each type of market, detection rates for H7N9 virus and for all AIVs and isolation rates for H7N9 virus quickly rebounded to preclosure or higher levels after the markets reopened, except for H7N9 virus detection and isolation rates in retail markets (Table 2). Detection rates for H7N9 virus and for all AIVs on chopping boards in retail LPMs were considerably higher than rates for other sampling sites (Table 2). During the study period, H7N9 virus was isolated from 23 environmental samples by virus culture. Of the 23 samples, 19 were collected in the nonintervention period, including those collected from all sampling sites in retail LPMs (Table 3). Only 4 virus-positive samples were identified during the market closure period (Figure 3); 3 were collected on the first day. The 4 positive samples were collected from all 3 types of poultry markets; 2 were from wastewater, 1 from a chopping board, and 1 from a defeathering machine.

**Table 3:** Influenza A(H7N9) virus identified in or on different environmental sites in 5 poultry markets under enhanced surveillance, Guangzhou, Guangdong Province, China, 2014*

Environmental sites	No. samples/no. tested (%)

Retail LPMs			Wholesale LPM			DPM	
rRT-PCR–positive	Culture-positive†	rRT-PCR–positive	Culture-positive†	rRT-PCR–positive	Culture-positive†
Before market closure and after market reopened								
Poultry cage	12/94 (12.8)	2/9 (2.8)		7/56 (12.5)	‡		–	–
Defeathering machine	11/59 (18.6)	2/10 (3.7)		4/30 (13.3)	1/3 (4.4)		–	–
Chopping board	18/95 (18.9)	6/14 (8.1)		–	–		4/26 (15.4)	0/1
Processing table	25/189 (13.2)	4/18 (2.9)		–	–		6/60 (10.0)	0/4
Bucket holding poultry meat	–	–		1/29 (3.4)	‡		–	–
Wastewater	13/95 (13.7)	2/11 (2.5)		10/80 (12.5)	2/3 (8.3)		3/34 (8.8)	0/2
Drinking water	–	–		1/6 (16.7)	‡		–	–

During market closure								
Poultry cage	2/91 (2.2)	0/2		3/59 (5.1)	0/3		–	–
Defeathering machine	4/58 (6.9)	0/4		2/30 (6.7)	1/2 (3.3)		–	–
Chopping board	9/96 (9.4)	1/8 (1.2)		–	–		3/27 (11.1)	0/3
Processing table	7/188 (3.7)	0/7		–	–		0/59	–
Bucket holding poultry meat	–	–		0/30	–		–	–
Wastewater	4/92 (4.3)	1/4 (1.1)		3/83 (3.6)	0/3		1/33 (3.0)	1/1 (3.0)
Drinking water	–	–		1/6 (16.7)	0/1		–	–

*DPM, dressed poultry market; LPM, live poultry market; rRT-PCR, real-time reverse transcription PCR; –, no samples collected. †Because not all positive samples from rRT-PCR were available for virus culture, isolation rates were derived by using the product of the percentage of rRT-PCR–positive samples and the percentage of those samples that were also culture-positive.  ‡These positive samples from rRT-PCR did not have sufficient material available for virus culture.

**Figure 4:**
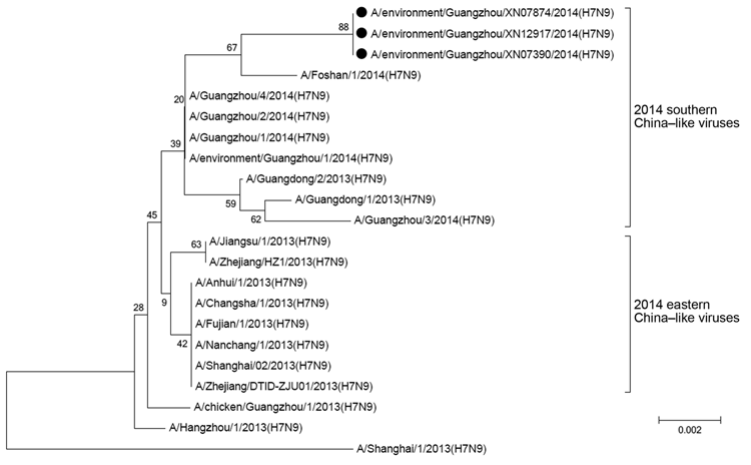
Phylogenetic analysis of hemagglutinin genetic sequences of an influenza A(H7N9) virus isolated from an environmental sample collected at a retail live poultry market under enhanced surveillance, Guangzhou, China, 2014. Black circles indicate 3 strains collected before (February 13), during (February 15), and after (March 17) a 2-week market closure. Scale bar indicates base substitution per site.

We sequenced 3 H7N9 virus strains collected from a the same enhanced surveillance retail LPM before, during, and after the market closure. All 3 strains had identical HA genes and differed from a strain collected on January 12 during routine surveillance, A/environment/Guangzhou/1/2014(H7N9), by mutations at 3 sites (D264E, R364K, and K414T). Phylogenetic analysis showed that the strains were genetically closer to the lineage in southern than in eastern China (Figure 4).

## Discussion

We report RNA detection rates for H7N9 virus and for all AIVs as well as viable virus survival in retail and wholesale LPMs and a DPM in Guangzhou before, during, and after market closure and disinfection. Before interventions were implemented on February 15, 2014, detection rates were much higher in retail markets than wholesale markets; this finding is in keeping with the theory that AIVs amplify in retail LPMs (*15*). During normal trading days, H7N9 virus was isolated in retail LPMs from numerous sampling sites, ranging from poultry cages at the back of the retail stalls to the processing tables and chopping boards near the customers. This finding indicates that poultry workers and customers had constant exposure to H7N9 virus in winter, when virus activity is high. 

H7N9 virus was infrequently identified on poultry farms after its emergence in 2013. For example, no H7N9 virus was detected in Guangdong farms until March 2014 (*16*), well after the first reported local case in a person in August 2013 (*17*). Infection with highly pathogenic H5N1 virus caused severe symptoms in poultry, but infection with low pathogenic H7N9 virus resulted in mild symptoms; thus, the poultry industry had little incentive to identify H7N9 virus–infected poultry. This fact may preclude efficient surveillance on poultry farms. Because the rate of H7N9 virus detection in retail markets is higher than that on poultry farms, LPMs may serve as a better surveillance point for AIV H7N9 virus. 

Our findings suggest that chopping boards and wastewater are more sensitive than other LPM environmental sources for the surveillance of AIV activity; this finding is consistent with those of others (*18*,*19*). We also isolated H7N9 virus in defeathering machines in wholesale LPMs. Surveillance programs and disinfection efforts should prioritize these environmental sources of virus contamination. These findings highlight the need to review and strengthen cleaning and disinfection procedures.

After the markets’ initial cleaning and disinfection at the commencement of the citywide market closure, RNA for H7N9 virus and for all AIVs were detectable throughout the 14-day market closure period, albeit at lower detection rates than before cleaning and disinfection. However, viable virus could be cultured only from samples collected within 2 days of market closure. This finding demonstrates that detection of viral RNA by rRT-PCR does not necessarily mean presence of infectious virus. Two of the 4 virus isolates obtained after the market closure were collected from wastewater rather than from solid dry surfaces. AIV can survive much longer (≥2 days) in water (*20*) than on environmental surfaces, and LPM workers who clean water containers may have a higher risk for AIV infection (*21*). Thus, wastewater must be removed or efficiently disinfected, and drinking water used by poultry must be removed if interventions such as those used in Guangzhou are to be effective. 

We did not quantify the infectious virus load by titration, even when virus could be cultured at the first sampling time point after market closure; thus, it is possible that the virus titer had decreased compared with that during the preintervention period. It is not clear whether virus titers would have been sufficient to initiate reinfection of reintroduced naive poultry. Previous studies on H9N2 virus demonstrated substantial reduction in isolation rates after a ban on keeping poultry overnight at LPMs, suggesting that transmission can be interrupted by emptying the market overnight (*22*). 

We did not collect samples during days 3–5 days after implementation of the interventions. However, the estimated reduction in H7N9 virus and other AIV RNA detection rates by rRT-PCR within a day of the citywide market closure was ≈50% from enhanced and routine surveillance (Figure 2).

We observed that all 3 types of poultry markets were recontaminated by H7N9 virus and other AIVs; immediately after markets were reopened, detection rates were as high as those before market closure. In contrast to the preintervention period, during the postintervention period, we found no difference in virus detection rates in the retail and wholesale markets. The reason for this is unclear. It is possible that the sudden closure of the LPMs led to a backlog of poultry in other temporary holding facilities, where mixing of poultry originating from different areas could have led to virus amplification before the poultry reentered the wholesale market. Aside from within-market interventions, tightening controls on poultry or shortening transportation time along the supply chain may be needed to further reduce virus load in LPMs. For the DPM, poultry may have been stored temporarily and prepared elsewhere to preserve freshness, which may have contributed to the unexpectedly high detection rates in this market.

Market rest days, along with a series of other control strategies, have been shown to reduce circulation of low pathogenic AIV in the retail LPM setting (*22*). Compared with other species commonly traded in LPMs (i.e., ducks, geese, and pigeons), chickens and quail were found to be more susceptible to H7N9 virus and shed higher levels of virus for a longer period (*23*). Because they are more susceptible to H7N9 virus, segregating chickens and quail from other species may limit virus transmission in retail and wholesale markets. More studies are needed to understand why LPMs were contaminated by H7N9 virus soon after they were reopened. If recontamination was due to off-site holding of multiple consignments of poultry in ad hoc storage areas, measures must be taken to minimize the need for such storage; well planned, preemptive interventions should replace reactive ones to which the poultry industry cannot rapidly adjust.

Although market closure has been demonstrated to be effective in reducing influenza infections in humans in China (*10*,*11*), its frequent or prolonged implementation may not be sustainable for the poultry industry, even if limited to winter when AIV activity is high. Furthermore, H7N9 virus RNA can also be detected in LPMs during summer (*24*), and such detections may trigger market closures. The general public in China tends not to favor the centralized slaughter of poultry, especially because poultry workers, whose income is disrupted and who experience other economic losses when markets are closed, object to the idea (*25*). Whether an approach that includes interventions such as species segregation, stringent testing at the wholesale market level, frequent cleaning and disinfection of markets, and regular market rest days may reduce the infection risk to a minimal but sustainable level remains to be investigated (*22*,*26*).

Poultry workers in China still demonstrate relatively low awareness of the risk for H7N9 virus infection and compliance with measures to prevent virus transmission (*27*). A serologic study in southern China showed that 54% (52/96) of poultry workers had seroconverted for H7N9 virus during May–December 2013 (*28*), although few cases were virologically confirmed. Among persons in whom cases of H7N9 infection were laboratory confirmed, >50% (43/84) had visited LPMs but only 5% (6/123) had occupational exposure to poultry (*29*). Risk for infection with H7N9 virus for the general public seems to be different from that for poultry workers, who have prolonged and direct exposure to poultry. Such discordance between potential exposure and disease is also noted with H5N1 virus infection and may reflect heterogeneity of host susceptibility to infection with these AIVs (*30*). Although limited human-to-human transmission of H7N9 virus among close contacts has been reported (*14*,*31*), the main transmission route seems to be associated with exposure to LPMs. Hence, special attention should be paid to the LPM environment, which provides the interface between poultry and the general public. For example, chopping boards, which are usually located at the front of the retail stalls, had higher isolation rates of H7N9 virus. Interventions such as adding a screen between customers and the chopping board may reduce the public’s exposure to H7N9 virus.

Our study has some limitations. First, the unexpected timing of the poultry market closure shortened the preintervention baseline period and the enhanced surveillance period during which we could obtain samples from LPMs. Thus, the preintervention data may not fully reflect the normal situation, especially in wholesale markets (Figure 3). However, enhanced surveillance showed consistent and substantial reductions in H7N9 virus detection in retail LPMs and the DPM as estimated from those before and after disinfection by routine surveillance. The estimated effect of market closure on H7N9 virus activity should be unbiased and most relevant to the general public, who are primarily exposed to poultry at retail LPMs and DPMs. Second, environmental and poultry samples were not collected in parallel and along the supply chain; thus, we could not identify potential interactions between retail and wholesale markets during the closure period or between poultry and the market environment during trading days. Whether the unexpected market closure led to unusual holding of poultry off site and whether this may have contributed to the postintervention rebound of virus in the markets is unclear. If such interventions are well planned and anticipated, poultry farmers can adjust their shipments to the wholesale market, and this rebound may be avoidable. Third, detection of H7N9 virus RNA does not directly translate into risk for transmission of the virus; transmission depends on multiple factors, such as virus viability (as assessed by virus isolation), infectious virus load, and mode and frequency of contact with different market environments. We did examine H7N9 virus isolation and RNA detection from different sampling sites and provide an overview of H7N9 virus contamination at different time points after market closure and from different environments, which supply an evidence base for fine-tuning current market interventions.

We document the effect of market closures on survival of H7N9 virus in a natural LPM setting. Market closure and disinfection reduced H7N9 viral RNA contamination in the LPM environment by >70% and infectious virus by >90%. However, live virus could be detected for ≈2 days after the intervention, especially in wastewater sources, and H7N9 virus activity returned quickly to preintervention levels once markets reopened. The reason for this rebound requires further investigation to inform the design of more effective interventions. Given limited support from the general public for permanent closure of LPMs (*32*), more sustainable alternative approaches should be considered to minimize the risk of transmission of H7N9 virus from retail LPMs. These approaches might include improving the design of retail stalls, segregating or banning poultry species with high susceptibility to AIVs, scheduling market rest days so that poultry farmers can adjust shipments, and improving viral surveillance. At the same time, unintended consequences of interventions, such as unauthorized movement of poultry from a closed market to a different trading area, should be avoided (*33*). To strike a balance between minimizing the risk of virus transmission to humans and the demands for live poultry from the public and the interests of the poultry industry, public health and veterinary sectors should strengthen their coordination under a One Health approach (*34*). Clarification of H7N9 virus prevalence along the poultry supply chain (from farm to retail markets), identification of key settings for virus amplification, and characterization of poultry trading patterns during normal and epidemic periods with various interventions will help in preparing an optimal control strategy.
